# From mice to humans—divergent strategies for intestinal homeostasis and regeneration

**DOI:** 10.1002/1873-3468.70387

**Published:** 2026-06-19

**Authors:** Keiko Ishikawa, Shinya Sugimoto, Toshiro Sato

**Affiliations:** ^1^ Department of Integrated Medicine and Biochemistry Keio University School of Medicine Tokyo Japan; ^2^ Department of Gastroenterology Keio University School of Medicine Tokyo Japan; ^3^ Keio University Health Center Tokyo Kanagawa Japan

**Keywords:** colonic stem cell, fetal‐like reprogramming, intestinal stem cell, LGR5^+^ stem cell, organoid, regeneration, slow‐cycling stem cell, somatic mutation rate

## Abstract

The intestinal epithelium is maintained by stem cells that balance self‐renewal and differentiation to sustain homeostasis and enable regeneration after injury. Recent advances—including organoid culture, genome editing, orthotopic xenotransplantation, and somatic mutation–based analysis—have created new opportunities to investigate intestinal stem cell (ISC) dynamics in humans. These studies have revealed striking species‐specific differences: whereas mouse LGR5^+^ ISCs divide daily and are chemo‐sensitive, human colonic LGR5^+^ stem cells are predominantly slow‐cycling and chemo‐resistant. Consistent with this reduced cycling, human ISCs accumulate somatic mutations more slowly than those of mice. Across mammals, ISC proliferation rates inversely correlate with lifespan, a relationship thought to minimize mutation accumulation and reduce cancer risk, in line with Peto's paradox. Regenerative responses in both mice and humans can involve fetal‐like reprogramming driven by YAP and other signaling pathways, yet the extent of species differences in intestinal regenerative capacity remains unclear. This review synthesizes current insights into ISC kinetics, injury responses, and evolutionary adaptations, highlighting the need for human‐focused studies to bridge translational gaps and guide regenerative medicine strategies.

## Graphical abstract

Recent advances such as organoid genome editing, xenotransplantation, imaging, and whole‐genome sequencing have enabled direct studies of human intestinal stem cells (ISCs). These studies reveal species‐specific features, including slower ISC proliferation, distinct injury responses, slower somatic mutation accumulation in humans, and an inverse relationship between mutation rates and lifespan across species, highlighting the importance of human‐oriented ISC research.

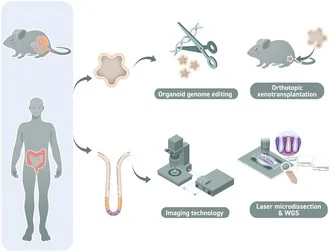

## Abbreviations

**BEST4**, Bestrophin 4

**BMP**, Bone morphogenetic protein

**CBC**, Crypt base columnar

**DCS**, Deep crypt secretory

**DSS**, Dextran sulfate sodium

**ECM**, Extracellular matrix

**EDTA**, Ethylenediaminetetraacetic acid

**EGF**, Epidermal growth factor

**FAK/Src**, Focal adhesion kinase and steroid receptor coactivator

**FGF‐2**, Fibroblast growth factor 2

**FKBP**, FK506 binding proteins

**IGF‐1**, Insulin‐like growth factor 1

**ISC**, Intestinal stem cell

**LGR5**, Leucine‐rich repeat‐containing G protein‐coupled receptor 5

**LRC**, Label‐retaining cell

***Ly6a***, Lymphocyte antigen 6 complex a

**NMR**, Naked mole rat

**PGE2**, Prostaglandin E2

**REG4**, Regenerating islet‐derived family member 4

**RXR**, Retinoid X receptor

***Sca1***, Stem cell antigen‐1

**TA**, Transit‐amplifying

**TGF‐β**, Transforming growth factor beta

**WGS**, Whole‐genome sequencing

**YAP**, Yes‐associated protein

## Rapid self‐renewal of the intestinal epithelium

The intestinal epithelium is organized into numerous crypt‐villus units (Fig. 1A–C), and this complex architecture underlies its two primary functions: efficient absorption of nutrients and protective barrier against external insults. The villus is a finger‐like protrusion of the gut wall that lines the lumen to increase the surface area for uptake of nutrients, which is covered by fully differentiated cells (Fig. 1B) [1]. At the base of the villus are crypts of Lieberkühn, or simply crypts, which are invaginations where LGR5^+^ crypt base columnar (CBC) cells reside at their bottom. Villus length decreases progressively toward the distal end of the intestinal tract. Unlike the small intestine, the colon lacks villi and instead contains densely packed, vertically aligned tubular crypts that form a flat luminal surface (Fig. 1C). The villi and crypt tops are continuously exposed to diverse external stimuli, including mechanical stress, microbial interactions, and dietary components. Consequently, these epithelial surfaces undergo rapid self‐renewal every 3–5 days to preserve tissue integrity and maintain functional homeostasis.

**Fig. 1:**
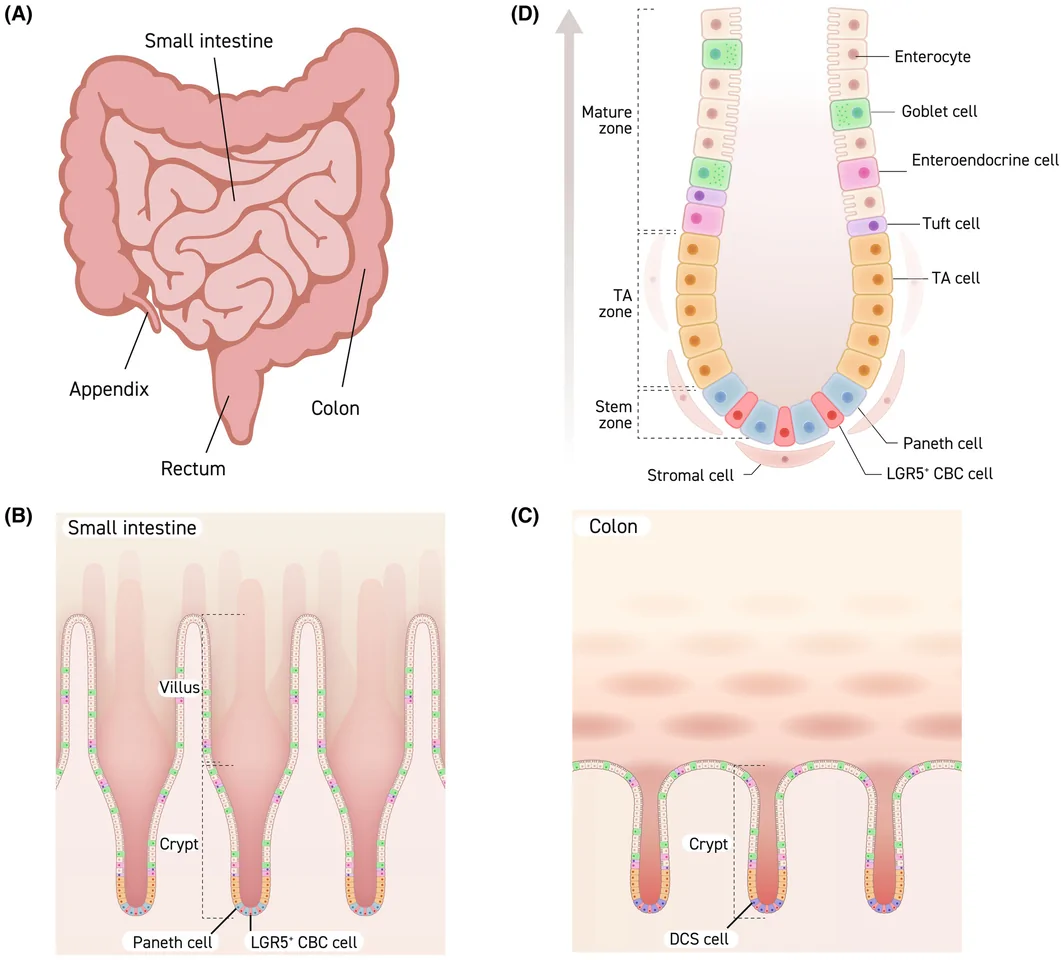
Intestinal structure. (A) Schematic model of the human intestine and colon. (B) The small intestinal epithelium is comprised of millions of crypt‐villus units. (C) The colonic epithelium lacks villi and the luminal surface is flat. (D) Current mouse model of stem cell hierarchy in the small intestine: LGR5^+^ crypt base columnar (CBC) cells interspersed with Paneth cells proliferate daily to generate TA cells and lineage‐committed progenitors. As LGR5^+^ CBC cells divide, their progeny migrates upward toward the crypt top or villus.

Stem cells are defined by their self‐renewal capacity, multi‐differentiation, and longevity [2]. Intestinal stem cells (ISCs) are supported by their microenvironmental niche, such as Paneth cells [3] and the mesenchymal cells [4, 5, 6, 7, 8, 9], which secrete growth factors, including WNT, R‐spondin, epidermal growth factor (EGF), NOTCH, and bone morphogenetic protein (BMP) ligands. ISCs, typically marked by LGR5 expression, continuously divide to give rise to progenitor cells and transit‐amplifying (TA) cells [1] (Fig. 1D). These progenitor cells eventually produce all mature epithelial lineages—enterocytes, goblet cells, enteroendocrine cells, Paneth cells, M cells, and tuft cells—as well as bestrophin 4 (BEST4)^+^ cells, which are absent in mice [10]. As these progenitors migrate upward along the villi or crypt tops, they undergo differentiation and are eventually shed from the epithelium through apoptosis, thereby maintaining crypt homeostasis [11]. LGR5^+^ cells represent ISCs but may also include progenitors. LGR5‐reporter analysis, and more recently, single‐cell transcriptomic profiling have identified two distinct clusters within the LGR5‐expressing population: an LGR5^high^ population enriched for CBC cells and an LGR5^low^ population enriched for absorptive and secretory lineage markers, indicative of TA cells [12, 13]. The colon lacks Paneth cells; instead, similar regenerating islet‐derived family member 4 (REG4)^+^ deep crypt secretory (DCS) cells occupy the crypt bottom [14]. Although epithelial turnover in the colon is slightly slower than in the small intestine [15, 16], both regions exhibit similar cellular dynamics in maintaining homeostasis and responding to injury.

## Insights from classical pulse‐chase studies into species‐specific ISC dynamics

### Cell‐cycle kinetics in the mouse intestine

Research on ISCs has been actively pursued since the 1950s, even before the advent of genetically modified mouse models, using histological analyses and thymidine analog‐based labeling experiments [17, 18]. Two competing hypotheses were proposed: the cycling CBC cell model by Cheng and Leblond [19], and the quiescent +4 cell model by Potten [15, 20]. CBCs were initially identified as radioactively labeled cells capable of giving rise to multiple epithelial lineages [19]. Subsequent approaches, including chemical mutagenesis labeling [21, 22] and genetic lineage tracing, further substantiated their stem cell identity. Chemical mutagenesis labeling demonstrated the emergence of long‐lived clonal ribbons over several months, encompassing multiple lineages and retaining at least a single CBC cell [22]. It is now widely accepted that LGR5^+^ CBC cells, intermingled with Paneth cells at the crypt bottom, represent the actively cycling stem cell population responsible for maintaining intestinal homeostasis [23]. Even before the development of genetic tracing techniques, studies had shown that mouse intestinal epithelial cells at the crypt base undergo a cell cycle of approximately 24 h; the S phase occupies approximately 5–8 h, while the combined G_2_ and M phases together span about 1–2 h [15, 24, 25, 26, 27]. These earlier observations are consistent with current views suggesting that LGR5^+^ ISCs proliferate daily in the mouse intestine [23, 28, 29].

Quiescence has traditionally been considered a defining feature of stem cells. In the G_0_ phase, cells that are in reversible arrest are termed quiescent, whereas those in irreversible arrest are classified as senescent or differentiated [30]. For instance, hematopoietic and muscle stem cells typically reside in this nonproliferative G_0_ state, which limits cell division, reduces replication errors, and prevents proliferative exhaustion. This mechanism safeguards their long‐term maintenance and functional integrity. In contrast, quiescence is not a universal hallmark of stem cells in rapidly renewing tissues, such as the intestinal epithelium.

Historically, studies on stem cell kinetics employed *in vivo* administration of thymidine, which posed ethical constraints and limited such analyses primarily to animal models or *ex vivo* systems. Thymidine analogs are incorporated into newly synthesized DNA during the S phase and become progressively diluted with each cell division. Cells that retain these labels—referred to as label‐retaining cells (LRCs)—were originally conceptualized based on the immortal strand hypothesis [20], which proposed that stem cells selectively retain their original DNA strands to minimize mutation accumulation. However, lineage‐tracing studies have challenged this concept, showing that LGR5^+^ ISCs exhibit random and symmetric DNA segregation with high turnover rates [28, 29, 31]. Early reports that LRCs are rare in the mouse intestine supported the once‐popular reserve stem cell model, in which infrequent LRCs or +4 cells serve as a backup for regeneration, whereas LGR5^+^ ISCs maintain homeostasis. However, the degree to which stem cell dynamics are conserved between species remains unclear, and caution is warranted when extrapolating mouse‐derived concepts to humans. In particular, stem cell kinetics in the human intestine are poorly characterized, and detailed assessments of stem cells or LRCs in humans are scarce. LRCs in mice and humans are unlikely to have identical roles or significance. In the following section, human LRCs are considered solely as markers of cellular quiescence, rather than indicators of reserve stem cell identity.

### Cell‐cycle kinetics in the human colon

Classical studies have shown that intestinal epithelial cell‐cycle dynamics vary across animal species and differ between anatomical regions of the intestine [32, 33, 34]. Unlike in mice, where LRCs are absent or extremely rare, LRCs have been identified in the human colon [35]. Due to a lack of specific genetic markers, distinguishing bona fide stem cells from committed progenitors was challenging. Furthermore, slow incorporation of DNA labels, together with the requirement for long chase periods, has made it difficult to estimate cell‐cycle duration of human ISCs accurately. Nonetheless, available findings suggest that human colonic stem cells exhibit a slower proliferative rate than their mouse counterparts. In one study, thymidine was intravenously administered to patients with colorectal cancer, and epithelial cell‐cycle kinetics were analyzed in histologically normal colonic tissue distant from the tumor [35]. LRCs were predominantly located at positions +0 to +5 from the crypt base. When the number of LRCs was plotted against chase duration, the curve displayed an inflection point, indicating the presence of a slowly proliferating cell population within human colonic crypts [25, 35]. The half‐life of these slow‐cycling cells was estimated to be at least 8 days, likely to reflect the intrinsic cell‐cycle characteristics of human colonic stem cells. However, because this analysis was performed on tissue from cancer patients, the findings may not fully represent the dynamics of completely normal human colonic epithelium. In another intriguing study, the tritiated thymidine labeling index at each cell position within the colonic crypt revealed apparent differences between mouse and human colons [16]. Notably, the human colon exhibited a markedly lower labeling index at the very base of the crypt, indicating minimal proliferative activity in this region compared with mice. A separate estimate placed the human ISC division rate at approximately once every 132–264 h [36]. By contrast, several earlier studies reported shorter cycle times (~ 48 h) for lower crypt cells, but these likely failed to distinguish ISCs from TA cells, which may have underestimated true stem cell‐cycle length [37, 38, 39]. Although direct comparisons are complicated by interspecies differences in crypt length and total cell number, these findings collectively suggest fundamentally different proliferative behavior at the crypt base.

Quantitative evaluation of LRCs can be influenced by multiple variables, including the intensity of the pulse label, the duration and timing of the pulse‐chase protocol, and the morphological criteria used to distinguish secretory cells from stem cells. Additional confounders, such as preoperative fasting, weight loss, consumption of a low‐residue or low‐fiber diet, and procedures of repeated endoscopies/biopsies, may also have affected cell proliferation rates in these studies [40].

## Genetic lineage tracing reveals species‐specific ISC dynamics

### Rapidly dividing mouse LGR5 ^+^ ISCs maintain intestinal integrity under homeostasis

While authentic stem cell identification requires prospective analysis, DNA analog‐based tracing has limitations in labeling specific cell types. To overcome this, a genetic lineage‐tracing system was developed in which LGR5^+^ ISCs were marked with Cre‐ERT2, and their progeny were visualized via a Cre‐reporter upon tamoxifen treatment [23]. As LGR5^+^ ISCs divide and differentiate, the label is inherited by all descendants, forming clonal ribbons extending from the crypt base. The long‐term persistence of these ribbons demonstrated both multipotency and self‐renewal capacity. Subsequent studies have provided definitive evidence that LGR5^+^ ISCs proliferate daily [23, 28, 41], and most LGR5^+^ ISCs are Ki67 positive [42], consistent with high proliferative activity of ISCs. *In vivo* live imaging of the mouse small intestine further confirmed that LGR5^+^ ISC cells divide daily, with those located at crypt base more likely to remain anchored within the niche [43]. ISCs compete for limited niche space via neutral drift, whereby cells randomly occupy available positions, ultimately leading to clonal dominance [31, 44].

The development of mouse small intestinal organoid culture has enabled long‐term *in vitro* maintenance of ISCs by providing niche factors consisting of R‐spondin, EGF and noggin [45]. Stem cell division and fate decisions are regulated by both cell‐intrinsic programs and extrinsic niche‐derived signals [46]. Multiple factors influence ISC proliferation, including obesity, fasting, gut microbiota, circadian rhythm and aging [47, 48, 49]. Obesity promotes ISC self‐renewal and tumorigenesis through nutrient‐sensing mechanisms, metabolic rewiring, and modulation of pathways such as peroxisome proliferator‐activated receptor signaling pathways and fatty acid oxidation [50, 51, 52, 53, 54]. In contrast, dietary interventions, such as caloric restriction, short‐term fasting, and ketogenic diets enhance ISC function [55, 56, 57, 58, 59]. A recent study showed that refeeding after short‐term fasting boosts ISC activity and regeneration but also facilitates tumor initiation via increased protein synthesis [60].

Circadian rhythm also regulates proliferation in the intestinal epithelium with crypt cell proliferation in mice peaking during the nocturnal phase, although it remains unclear whether this applies exclusively to LGR5^+^ ISCs [61, 62, 63]. Gut microbial metabolites, such as butyrate, a short‐chain fatty acid, can suppress ISC proliferation [64]. Age‐related decline in ISC function is partly due to altered niche‐derived signals; aged crypts contain fewer LGR5^+^ ISCs than young crypts, though their proliferation rate remains unchanged [65]. In aged Paneth cells, elevated NOTUM expression suppresses WNT signaling, contributing to reduced ISC function and regenerative capacity. Because proliferation and stem cell dynamics are affected by variables, such as the anatomic region, time of tissue collection, light–dark cycles, and housing conditions (i.e., germ‐free versus conventional), there is an urgent need for standardized methodologies to assess ISC dynamics.

### Fetal‐like reversion triggers postinjury regeneration in mouse models

The reserve stem cell model, in which a small population of quiescent stem cells serves as a backup pool for epithelial regeneration after injury, was long accepted in mouse studies. Multiple + 4 markers, or quiescent stem cell markers, have been proposed, including *Bmi1* [66, 67], *Hopx* [68], *Tert* [69], *Lrig1* [70], and *Mex3a* [71]. This model posited that actively cycling ISCs are highly sensitive to injury, whereas quiescent reserve stem cells (including LRCs) are injury‐resistant and can gain stemness upon depletion of the cycling LGR5^+^ ISC population [72].

However, the current view is that dedifferentiation or cellular plasticity, rather than a distinct reserve stem cell population, represents the dominant and pervasive mechanism underlying intestinal regeneration. Firstly, the reserve stem cell population is too small to fully account for the highly dynamic process of intestinal regeneration [73]. Actually temporal lineage tracing and LGR5^+^ ISC ablation experiments demonstrated regenerated LGR5^+^ ISCs arise mainly from committed progenitors of absorptive and secretory lineages in an ASCL2‐dependent manner, supporting the idea that ISC regeneration is driven primarily by dedifferentiation [74]. Moreover, so‐called +4 markers are not specific to reserve stem cells, as they are also expressed in LGR5^+^ ISCs and various lineage‐committed progenitors [12, 75, 76, 77, 78, 79].

Although the underlying mechanisms differ, a variety of injuries such as irradiation, chemotherapy, and dextran sulfate sodium (DSS)‐induced colitis all result in depleting cycling LGR5^+^ ISCs. Irradiation and chemotherapy primarily eliminate LGR5^+^ ISCs through DNA damage and replication stress, whereas DSS‐induced colitis ablates these cells mainly through epithelial barrier disruption and immune‐mediated cytotoxic responses. Tian *et al*., [66] demonstrated that crypt homeostasis can be preserved even after ablation of LGR5^+^ ISCs, suggesting that non‐ISC sources are recruited to the ISC compartment to restore the crypt‐villi architecture. However, a subsequent study using more persistent and robust LGR5^+^ ISC ablation strategies demonstrated that sustained LGR5^+^ ISC ablation compromises intestinal epithelial integrity, supporting an essential role for LGR5^+^ ISCs under physiological conditions [80]. Genetic lineage‐tracing studies have shown that secretory progenitors can regenerate LGR5^+^ ISCs after irradiation [81, 82] and absorptive progenitors can generate new LGR5^+^ ISCs following LGR5^+^ ISC ablation [83]. The fully differentiated cells, such as enteroendocrine cells and Paneth cells, can revert to a stem cell fate after irradiation damage [79, 84]. Similar secretory‐to‐stem reversion has been observed in the mouse colon using DSS‐induced colitis or LGR5^+^ ISC ablation [85, 86, 87].

Clonal lineage tracing combined with irradiation injury and single‐cell trajectory analysis identified a transient ‘revival stem cell’ state that emerges during regeneration [88]. These cells express fetal‐associated markers such as clusterin (*Clu*), annexin A1 (*Anxa1*), and lymphocyte antigen 6 complex a (*Ly6a/Sca1*), originate from LGR5 lineage progeny, and reconstitute the ISC pool, supporting a model in which ISC regeneration is driven by dedifferentiation. Notably, the transcriptional profile of ‘revival stem cells’ resembles that of fetal intestinal epithelium, suggesting that regenerative dedifferentiation involves reactivation of a fetal‐like gene expression program. Nusse *et al*., [89] firstly established the concept that injury, such as *Heligmosomoides polygrus* infection, induces a fetal‐like transcriptional program in the intestinal epithelium. Subsequent studies identified the activation of fetal‐like transcriptional programs and Hippo/YAP signaling as a primary regenerative mechanism [88, 90, 91]. In the DSS‐colitis model, fetal‐like reprogramming is accompanied by extracellular matrix (ECM) remodeling, particularly increased collagen I deposition, which activates integrin‐focal adhesion kinase and steroid receptor coactivator (FAK/Src) signaling and subsequent YAP/TAZ‐mediated reprogramming [92]. Several studies have highlighted prostaglandin E2 (PGE2) as an important upstream regulator of YAP signaling [93, 94]. A recent paper identified mesenchyme‐derived asporin as a paracrine niche factor which facilitates fetal‐like reprogramming via transforming growth factor β (TGF‐β) signaling after chemotherapy‐induced injury [95]. TGFβ1 also promotes fetal‐like reprogramming following irradiation‐induced injury through YAP signaling [96]. Multiple candidate factors and pathways have been implicated in fetal reprogramming, many of which appear to converge on YAP signaling, suggesting a central role for YAP in driving fetal‐like transcriptional reprogramming.

Consequently, the classical reserve stem cell model has been revised to include newer concepts—dedifferentiation, revival stem cell, and fetal‐like reversion [74, 88, 91]. The exact cell types undergoing fetal reversion and the molecular ‘on/off’ switches governing this process remain an active area of investigation. For example, secretory cells expressing markers such as *Neurog3* and *p57* have been shown to dedifferentiate while acquiring fetal‐like transcriptional signatures [97, 98]. In intestinal organoid models, the transition from a fetal‐like state to a homeostatic epithelial identity is regulated by retinoid X receptor (RXR) signaling [99]. Upstream signals inducing YAP‐mediated fetal reprogramming are also being uncovered. However, fully recapitulating the niche conditions required for fetal‐like reversion *in vitro* remains challenging, and the detailed mechanisms controlling fetal reprogramming are still largely unknown. For a comprehensive overview of fetal reprogramming, readers are referred to specialized reviews [91].

### Emerging organoid technologies to investigate human ISC dynamics

Recent technological advances—including organoid genome editing, orthotopic xenotransplantation, imaging techniques, spontaneous somatic mutation–based tracing, and microdissection‐based whole‐genome sequencing (WGS)—have enabled the study of ISC dynamics in their native *in vivo* context not only in mice, but also in other species, including humans (Fig. 2A). Organoid technology enables the reconstruction of tissue architecture and function by providing niche factors that support stem cell self‐renewal in a controlled microenvironment [45]. Human small intestinal and colonic organoid culture methods were established by supplementing media with an activin‐like receptor kinase inhibitor and a p38 MAP kinase inhibitor [100]. A modified organoid culture method, substituting the p38 MAPK inhibitor with insulin‐like growth factor 1 (IGF1) and fibroblast growth factor 2 (FGF2), supports both epithelial self‐renewal and multi‐lineage differentiation, thereby recapitulating key features of ISC behavior *in vitro* [101]. Patient‐derived organoids can recapitulate disease‐specific tissue contexts when cultured under modified niche conditions, allowing modeling of pathogenic environments [102, 103]. By introducing defined genetic alterations, organoids offer a platform to study genotype–phenotype relationships in human genetic disorders. Moreover, genetic barcoding using CRISPR‐Cas9‐mediated engineered organoids enables gene knock‐in, knockout, and reporter tagging, facilitating applications such as genetic lineage tracing and targeted ablation of LGR5^+^ ISCs [104]. A recent study applied a split‐Cre system to human organoids for the first time, enabling simultaneous visualization of dual gene and lineage tracing specifically in cells co‐expressing both genes [105]. In this system, Cre recombinase was split into two fragments that reconstitute functional activity only when brought together via dimerizer‐induced FK506 binding proteins (FKBP)‐mediated heterodimerization. This split‐Cre strategy was originally developed by Buczacki *et al*., [72] These advances have enabled the construction of diverse disease models, contributing to the elucidation of underlying mechanisms and the development of novel therapeutic strategies. However, long‐term *in vitro* tracking of stem cells relies on abundant exogenous niche factors, creating conditions that differ significantly from the native *in vivo* environment and lack stromal interactions.

**Fig. 2:**
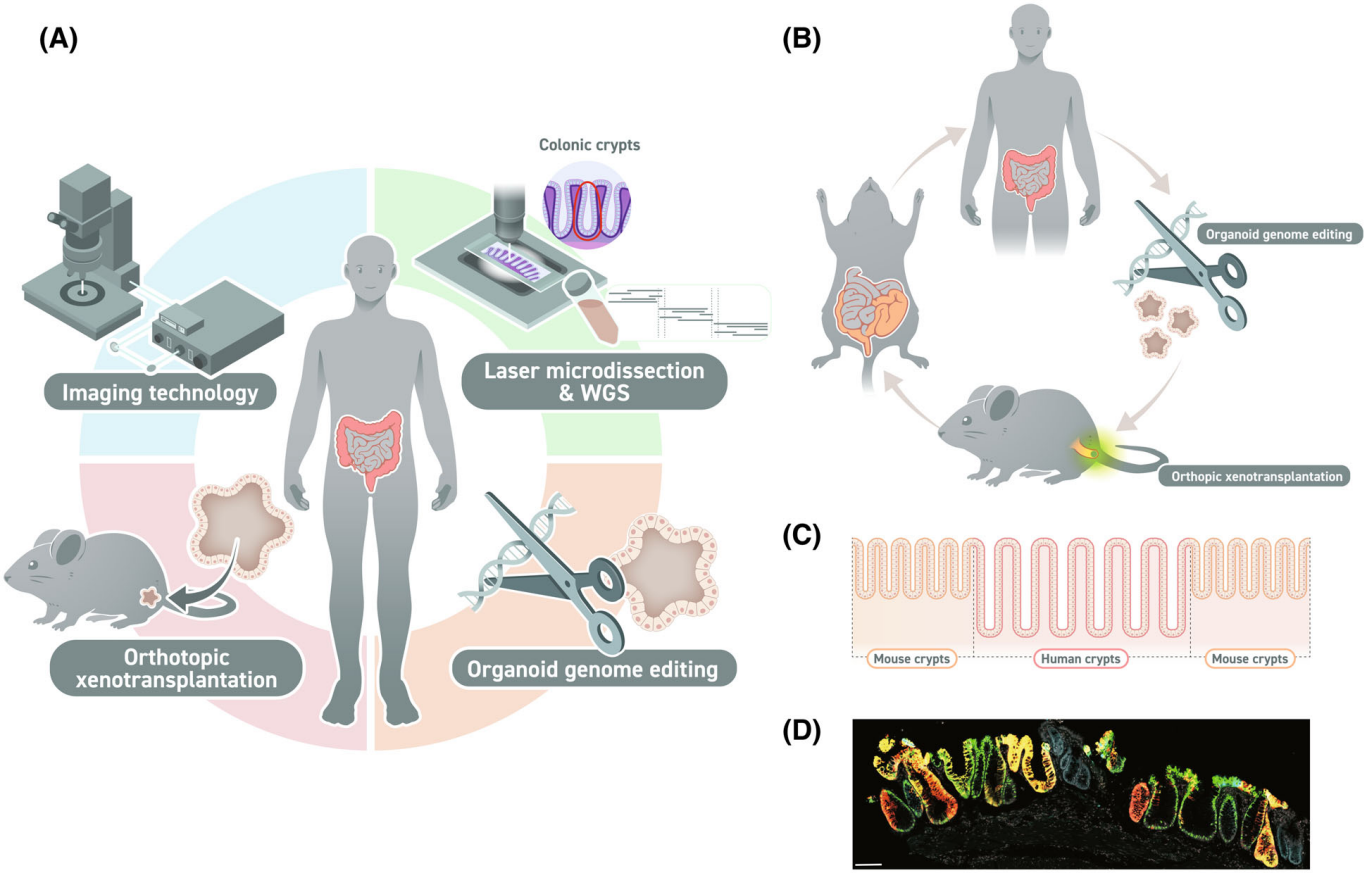
Advanced technologies for studying human investigate intestinal stem cell (ISC) dynamics. (A) A range of technological approaches to human ISC dynamics. Integration of whole‐genome sequencing (WGS) on microdissected tissues, organoid genome editing, orthotopic xenotransplantation, and advanced imaging technologies has opened new avenues for the analysis of human ISC dynamics. (B) Graphical overview of orthotopic xenotransplantation using genome‐edited human colon organoids. Successful engraftment is confirmed by colonoscopy through visualization of fluorescent‐positive areas. (C) Schematic representation of histological sections showing human engrafted crypts and adjacent native mouse crypts in an orthotopic xenograft model. Gene‐edited human colon organoids are xenotransplanted into the epithelium‐denuded colon of immunodeficient mice via the transanal route. The xenotransplanted human crypts are visibly larger than the adjacent native mouse crypts. (D) Confocal images of the LGR5 lineage tracing using human LGR5‐CreER/rainbow normal colon organoid xenograft. Nuclear GFP expression and LGR5‐rainbow reporter^+^ cells (red, yellow and cyan) are observed in the orthotopic xenograft on day 90 after tamoxifen treatment. Scale bar: 100 μm.

An important step toward physiological relevance has been the development of an orthotopic xenotransplantation model [106, 107, 108], which is achieved by transanal denudation of the distal colonic mucosa in immunodeficient mice using ethylenediaminetetraacetic acid (EDTA) and mechanical abrasion, followed by intraluminal delivery of human colonic organoids. Successful engraftment of human colonic organoids is assessed by fluorescent labeling and *in vivo* detection of these organoids using mouse colonoscopy at 1 month post‐transplantation (Fig. 2B–D) [108]. This platform permits near‐native reconstruction of human colonic epithelium and provides a powerful framework for direct functional analysis and lineage tracing of LGR5^+^ ISCs within a physiologically relevant context (Fig. 2B–D). To exclude the possibility of artificial accumulation of specific genetic abnormalities during organoid genetic engineering or xenotransplantation procedures, they compared RNA sequencing and WGS profiles of nonengineered organoids, postengineering organoids, and postxenotransplantation samples. No overt genetic or transcriptional abnormalities were detected [105]. While the mouse intestinal epithelium has served as the canonical model for mammalian ISC biology, the extent to which mouse ISCs differ from their human counterparts remains incompletely defined. Translating rodent findings into clinical applications therefore requires a precise understanding of both shared and species‐specific features of the intestinal mucosa. Human data, however, have historically been limited to static histological ‘snapshots’, restricting insights into ISC dynamics.

Recent imaging technologies, such as multiphoton microscopy, employ near‐infrared lasers to achieve deep tissue penetration with reduced phototoxicity. These approaches enable high‐resolution visualization of intestinal crypt architecture and single stem cell behavior prospectively in thick tissues and are particularly well suited for long‐term intravital imaging of ISC dynamics *in vivo* (Fig. 2A). Indeed, intravital imaging of colon cancer stem cells *in vivo* has recently been achieved through the combination of organoid gene engineering, xenotransplantation, and up‐to‐date imaging technologies [109]. However, applying this approach to human normal colon remains technically demanding.

Current WGS‐based approaches, in combination with clonally expanded organoid cultures, have enabled quantitative assessment of mutation burden in normal epithelia. Sequencing clonal organoid culture enables comprehensive characterization of mutational landscapes across multiple tissues, revealing age‐related mutation accumulation in normal colon [110]. Application of this approach to disease models has further provided mechanistic insights. For example, in patients with ulcerative colitis, an increased frequency of clones harboring mutations in genes associated with the IL‐17 signaling pathway has been observed, shedding light on disease pathogenesis and its contribution to colorectal tumorigenesis [111].

### *In vivo* ISC dynamics in the human colon: slow‐cycling and injury‐resistant

While genetic barcoding using engineered organoids provides artificial lineage markers, spontaneous somatic mutation–based analyses allow clonal lineage reconstruction using naturally accumulated mutations as endogenous barcodes. More recently, laser capture microdissection–coupled WGS has enabled spatially resolved mutation mapping directly *in situ* (Fig. 2A). Recent studies have revealed species‐specific differences in somatic mutation rates, crypt monoclonalization kinetics, and stem cell replacement rates between mouse and human colonic epithelium [112, 113, 114]. These findings consistently indicate that human ISCs cycle more slowly than those of mice. Combining orthotopic xenotransplantation with genetic lineage tracing has validated the stemness and functionality of human ISCs *in situ* and revealed the presence of LGR5^+^ Ki67^−^ cells in the human colon and xenografts—contrasting with the predominantly Ki67^+^ status of LGR5^+^ cells in the surrounding mouse colon [108]. Integrating transplantation, single‐cell transcriptomics, and organoid‐based lineage tracing has enabled detailed characterization of quiescent LGR5^+^ ISCs in the human colon, marked by p27, which divide approximately once per week [105]. In injury models, human LGR5^+^ ISCs showed resistance to chemotherapy‐induced damage, whereas adjacent mouse LGR5^+^ ISCs were sensitive, demonstrating chemoresistance in human LGR5^+^ ISCs. Lineage tracing confirmed that quiescent human ISCs contribute to homeostasis and expand their clonal output following injury, indicating a key role in postinjury regeneration. Thus, unlike the mouse intestine, the human colon appears to rely primarily on slow‐cycling stem cells for regenerative responses [105]. Notably, human intestinal epithelium also exhibits cellular plasticity and fetal‐like reprogramming, similar to that in mice, mediated by YAP [92] or TGF‐β‐YAP signaling [96].

For decades, functional interrogation of ISCs in living human tissues was largely impossible due to ethical and technical constraints. Recent advances now challenge the long‐standing mouse‐derived regeneration paradigm and highlight the importance of human‐focused ISC research. From another perspective, histology‐based lineage tracing offers only population‐level averages, whereas single‐cell‐level fate mapping via live *in vivo* imaging would yield higher spatiotemporal resolution. Such imaging has been achieved in the murine intestine [43] and in organoid‐based colorectal cancer xenograft models [109], but it remains technically challenging in the normal human intestine. Given the growing interest in intestinal regenerative medicine, bridging the knowledge gap between mouse and human ISCs is now a critical priority.

## Intestinal cell dynamics vary across species: stem cell‐cycling status and evolutionary biology

Each time a cell divides, somatic mutations inevitably accumulate. Larger, longer‐lived animals would therefore be expected to face a higher lifetime cancer risk than smaller, short‐lived species. Yet, this is not observed—an evolutionary puzzle known as Peto's paradox [115]. Two recent studies have provided mechanistic insights into this paradox.

In a comparative analysis of cancer incidence across 91 animal species, Vincze *et al*., [116] found no correlation between cancer risk and either body size or lifespan, reinforcing Peto's paradox. Cancer mortality represented substantial phylogenic variation, supporting the notion that cancer resistance has evolved independently across mammalian lineages. This paper also reported that high‐fat, low‐fiber diets are associated with increased cancer mortality, with carnivorous species exhibiting the highest cancer‐related death, indicating that ecological factors beyond lifespan and body size influence cancer susceptibility. Cagan *et al*., [117] performed WGS on microdissected colonic crypts from 16 mammalian species and discovered a strong inverse correlation between lifespan and annual somatic mutation rates. Long‐lived species accumulated far fewer mutations per year than short‐lived species, yet the total lifetime mutation burden at death was remarkably similar across species. This explains why various mammals have similar lifetime cancer incidence. The authors report that anticorrelation between lifespan and mutation burden is maintained for the three types of mutation signatures across species, indicating multiple biological process controlling this phenomenon. Although Cagan *et al*., provided partial resolution of Peto's paradox, another fundamental question remains unresolved. Long‐lived species are typically large‐bodied and thus composed of substantially more cells, which should theoretically increase the probability of malignant transformation. How such organisms suppress cancer risk despite this numerical disadvantage remains an open evolutionary and mechanistic question [118].

Although the slower cycling of human colonic stem cells may partially explain interspecies differences in mutation accumulation and tumorigenic potential, stem cell division rates alone are insufficient to account for the observed variation, based on prior estimates of stem cell division rates in mice, rats, and humans [117]. This discrepancy is likely attributable to species‐specific differences in DNA repair system, mutation rates per cell division, altered ISC dynamics and crypt architecture, and evolutionarily tuned cancer‐suppressive mechanisms. For example, elephants harbor multiple copies of the TP53 tumor suppressor gene, compared with single copy in humans, enhancing cancer suppression [119]. A recent study provided evidence for enhanced DNA repair capacity in the long‐lived, large‐bodied bowhead whale. The authors reported that bowhead whale fibroblasts and tissues exhibit high expression of the cold‐inducible RNA‐binding protein CIRBP, suggesting the presence of a species‐specific mechanism that contributes to improved genome integrity [120].

In long‐lived species such as the naked mole rat (NMR; > 25 years), LGR5^+^ ISCs proliferate more slowly than in short‐lived mice (~ 3 years) [121]. Despite being phylogenetically closer to mice than to humans, NMRs exhibit an intestinal turnover rate closer to that of humans. Although systematic cross‐species comparisons remain scarce, data from mice, NMRs, and humans suggest an inverse relationship between the organismal lifespan and true stem cell division rates [121] (Fig. 3). This relationship is absent when analyzing LGR5^+^ cells above the crypt base, which are likely TA cells, implying that evolutionary pressures primarily target long‐lived stem cell populations rather than progenitors [121].

**Fig. 3:**
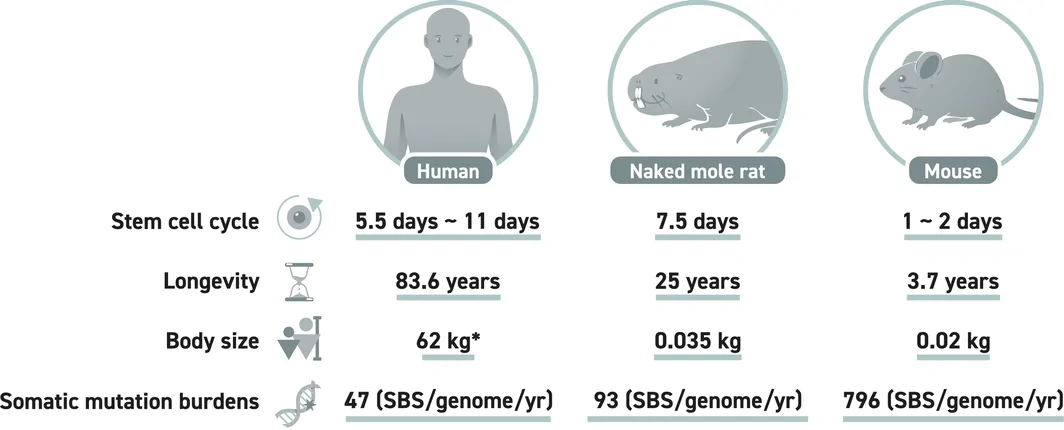
Distinctions in investigate intestinal stem cell (ISC) dynamics among humans, naked mole rats (NMRs) and mice. Shown are differences in stem cell‐cycling rates, longevity, body size and annual somatic mutation burdens across species. *A global anthropometric analysis estimated that the mean adult human body mass worldwide is approximately 62 kg, based on WHO BMI distributions and United Nations population data [126].

Prolonged stem cell survival carries a risk of mutation accumulation, yet organisms appear to have evolved strategies to minimize the fixation of such mutations, limiting age‐related dysfunction and tumorigenesis [122, 123, 124]. However, somatic mutation accumulation is unlikely to be the sole determinant of the aging process; additional mechanisms including telomere attrition and epigenetic alterations also play important roles. Elucidating human‐specific mechanisms that regulate the pace of aging will therefore be an important direction for future research. Long‐lived stem cells reduce replication‐associated mutations by conserving energy and remaining in a quiescent, cell‐cycle‐exited state for extended periods. The slower proliferation of human ISCs compared with those in short‐lived species such as mice likely reflects an evolutionary adaptation shaped by lifespan, body size, environmental pressures, and cancer suppression [46]. In essence, species have fine‐tuned stem cell proliferation rates to align with their life‐history traits, constraining somatic mutation accumulation and reducing cancer risk—potentially driven by yet unidentified selective pressures favoring longevity.

## Further perspectives

Recent technological advances have enabled the first meaningful steps toward analyzing ISC dynamics directly in the human context. Given the long lifespan and large body size of humans, the slow proliferative behavior of ISCs may represent an evolutionary adaptive strategy. While this slow‐cycling nature confers resistance to injury‐induced stress, a comprehensive understanding of human ISC kinetics remains elusive and warrants further investigation. Extending analyses of ISC dynamics to other large mammals beyond humans and mice will be important to enable more robust assessments of potential correlations between ISC dynamics and lifespan across species.

Notably, recent efforts have begun to establish cross‐species organoid platforms. For example, Kim *et al*., [125] generated diverse bat organoids from multiple organs and viral infection models to investigate infectious disease biology and healthy aging. The development of such systematic ‘cross‐species organoid zoo’ platforms could not only improve preparedness against zoonotic viral transmission but also enable the analysis of ISC dynamics in other large mammals that are phylogenetically closer to humans than mice. Combined with genome editing, cutting‐edge imaging technologies, and WGS, these systems will provide powerful opportunities to investigate the evolutionary and biological aspects of ISC dynamics across species.

Several key questions arise. Mechanistic control: What molecular and cellular mechanisms modulate the cell cycle in human intestinal epithelium? Model fidelity: To what extent do findings from orthotopic xenograft models—particularly those highlighting quiescent stem cells and injury resistance—reflect actual human physiology and clinical outcomes? Regenerative pathway: Which signaling networks and epigenetic reprogramming events drive regeneration in the human intestine? Interindividual variation: How do factors, such as age, sex, ethnicity, or disease state influence stem cell proliferation, renewal, and plasticity? Spatial heterogeneity: Are there positional differences in stem cell behavior within the crypt, and does stochastic entry into and exit from the niche compartment, as observed in mice, also occur in humans? Evolutionary context: What conserved molecular mechanisms across divergent species regulate stem cell proliferation and mutation rates, and to what degree does stem cell quiescence contribute to organismal longevity?

While mouse models remain indispensable for probing disease mechanisms, fundamental differences in stem cell dynamics and genomic integrity regulation between mice and humans underscore the importance of prioritizing human‐oriented research to bridge translational gaps.

## Author contributions

KI contributed to the conception, design, manuscript writing, figure design. SS contributed to the financial support, and manuscript revision. TS contributed to the conception, design, financial support, administrative support, manuscript revision, and final approval of manuscript.

## Conflict of interest

TS is an inventor on several patents related to organoid culture. The other authors declare no relevant conflicts of interest.
